# Antiemetic-associated QT prolongation: a clinical review and risk-stratified prescribing framework for long QT syndrome

**DOI:** 10.1093/ehjcvp/pvag077

**Published:** 2026-09-16

**Authors:** Ahmed T Moustafa, Omar Dabash, Pradosh Kumar Panigrahi, Habib Rehman Khan

**Affiliations:** London Health Sciences Centre Research Institute, Western University, London, Ontario N6G5A5, Canada; Schulich School of Medicine and Dentistry, Western University, London, Ontario N6G5A5, Canada; Royal College of Surgeons in Ireland, Dublin, D02YN77, Ireland; Schulich School of Medicine and Dentistry, Western University, London, Ontario N6G5A5, Canada; London Health Sciences Centre Research Institute, Western University, London, Ontario N6G5A5, Canada; Schulich School of Medicine and Dentistry, Western University, London, Ontario N6G5A5, Canada

**Keywords:** Long QT syndrome, Antiemetics, Torsades de pointes, QT prolongation, Ondansetron, Drug safety

## Abstract

**Aims:**

Nausea and vomiting accompany pregnancy, gastroenteritis, migraine, the perioperative period, and cytotoxic chemotherapy, and antiemetics are among the most frequently prescribed drugs worldwide. Several antiemetic classes delay ventricular repolarization and prolong the QT interval, a substrate for torsades de pointes (TdP), ventricular fibrillation, and sudden cardiac death. We synthesized the evidence on antiemetic QT liability and translated it into a three-tier, risk-stratified prescribing framework and stepwise clinical algorithm for patients with inherited or acquired long QT syndrome (LQTS) and other torsadogenic risk factors.

**Methods and results:**

We conducted a narrative review of PubMed/MEDLINE, EMBASE, and pharmacovigilance sources (FDA Adverse Event Reporting System, CredibleMeds/QTdrugs) through early 2026, prioritizing mechanistic studies, randomized comparisons, disproportionality analyses, and consensus statements on antiemetic QT liability and LQTS management.QT prolongation across antiemetic classes is driven principally by IKr blockade (hERG/KCNH2). Risk is highly heterogeneous within classes: in a 2026 FAERS disproportionality analysis, ondansetron carried a substantially stronger QT-related signal than olanzapine (reporting odds ratio 27.2 vs. 8.7), consistent with its dose-dependent effect among 5-HT3 antagonists, which prompted a 2012 FDA restriction on the 32 mg intravenous dose; palonosetron has minimal effect. Neurokinin-1 antagonists, low-risk antihistamines, anticholinergics, and 5-HT4 agonists carry little QT liability, while butyrophenones, phenothiazines, and promethazine carry greater risk. Serious events cluster in patients with electrolyte disturbance, bradycardia, polypharmacy, or underlying LQTS, particularly when QTc exceeds 500 ms or rises more than 60 ms from baseline.

**Conclusion:**

Antiemetic-associated arrhythmia is largely preventable. Structured risk assessment, electrolyte correction, ECG monitoring, and a stepwise selection strategy favouring agents with minimal QT liability allow clinicians to manage nausea and vomiting safely, even in high-risk populations.

## Introduction

Nausea and vomiting are among the most common symptoms in clinical practice, arising in pregnancy, gastroenteritis and other infections, migraine, motion sickness, the perioperative period, and during cytotoxic chemotherapy and radiotherapy.^1,2^ Beyond their effect on quality of life, severe or protracted vomiting causes dehydration and depletion of potassium, magnesium, and calcium, electrolytes that govern cardiac repolarization. Hence, the illness being treated may itself prime the heart for arrhythmia.^3^ Antiemetics are consequently prescribed frequently across oncology, anaesthesia, emergency medicine, obstetrics, and primary care, and structured guideline frameworks now govern prophylaxis for chemotherapy-induced nausea and vomiting (CINV) and postoperative nausea and vomiting (PONV).^4,5^ We searched PubMed/MEDLINE and EMBASE from January 2000 to February 2026 using combinations of the terms ‘antiemetics’,‘5-HT3 receptor antagonists’,‘dopamine antagonists’,‘long QT syndrome’,‘torsades de pointes’,‘QT prolongation’, and ‘drug-induced arrhythmia’, supplemented by hand-searching reference lists and relevant society guidelines; the full search strategy, terms, and inclusion/exclusion criteria are provided in the *Supplementary Material*.

A clinically important and underappreciated consequence of antiemetic use is delayed ventricular repolarization, manifest on the electrocardiogram (ECG) as prolongation of the QT interval. Marked QT prolongation can precipitate torsades de pointes (TdP), a polymorphic ventricular tachycardia that typically causes syncope when self-terminating but that can degenerate into ventricular fibrillation (VF), producing cardiac arrest and sudden cardiac death.^3^ Drug-induced QT prolongation has been a leading cause of post-marketing drug withdrawal and has shaped modern regulatory science, including the mandatory ‘thorough QT/QTc study’.^3,6^ The at-risk substrate is encountered constantly, and prolonged QTc is common among hospitalized and oncology patients and frequently coincides with electrolyte abnormalities and QT-prolonging drug exposure, with antiemetics among the most commonly implicated agents.^7,8^

Crucially, the magnitude of risk is not uniform across or even within antiemetic classes, and much of the observed harm is concentrated in patients with predisposing factors such as inherited LQTS, electrolyte derangement, bradycardia, structural heart disease, or concurrent QT-prolonging medications.^9,10^ This review revisits the relationship between antiemetics and QT prolongation through a contemporary, cardiology-focused lens grounded in the strongest available evidence. We summarize the physiology of repolarization and the molecular basis of congenital and acquired LQTS, examine the QT liability of each major antiemetic class with reference to key trials and pharmacovigilance studies, and translate this evidence into a risk-stratified prescribing framework, supported by a classification table and a stepwise clinical algorithm, intended to improve patient safety without compromising symptom control.

## The QT interval and ventricular repolarization

The cardiac action potential reflects the orchestrated movement of ions across the cardiomyocyte membrane and unfolds in five phases. Phase 0 (rapid depolarization) is driven by a fast inward sodium current; phase 1 (early repolarization) reflects transient outward potassium current; phase 2 (the plateau) balances inward L-type calcium current against outward potassium currents; phase 3 (rapid repolarization) is dominated by the delayed rectifier potassium currents, the rapid component (IKr, encoded by KCNH2/hERG) and the slow component (IKs, encoded by KCNQ1); and phase 4 restores the resting potential.^3,11^ The action potential phases, underlying currents, and their relationship to the surface ECG and QT interval are summarized in *Figure 1*.

**Figure 1:**
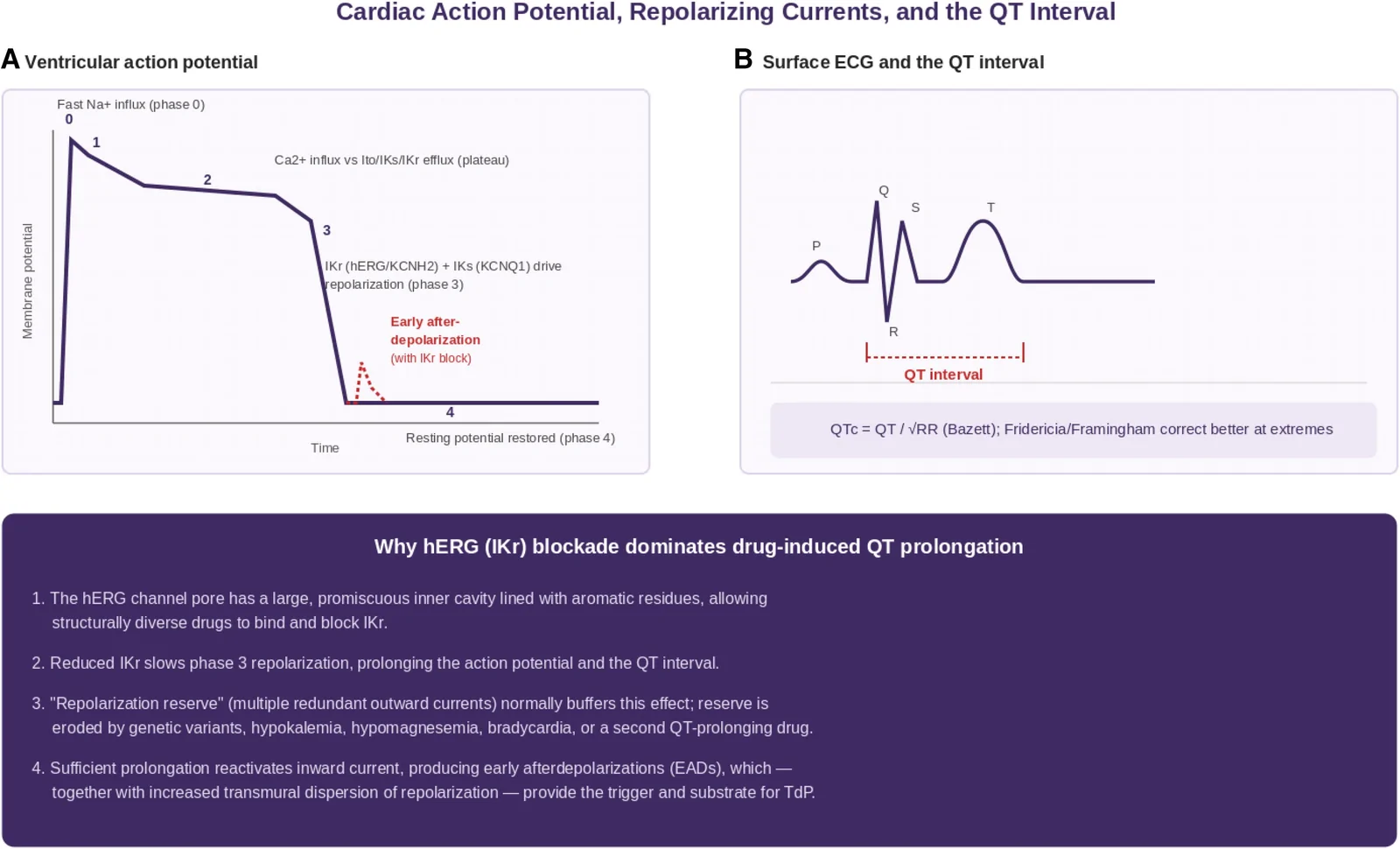
Cardiac action potential, repolarizing currents, and the QT interval. (*A*) The ventricular action potential phases (0–4) and the ionic currents that generate them; (*B*) The corresponding surface ECG and the QT interval. The lower panel summarizes why hERG (IKr) channel blockade is the dominant mechanism of drug-induced QT prolongation, including the concept of repolarization reserve and early after depolarizations (EADs) as the trigger for torsades de pointes.

Because the QT interval shortens as heart rate increases, it is conventionally corrected for rate (QTc). The Bazett formula is most widely used but over-corrects at high rates and under-corrects at low rates; the Fridericia and Framingham corrections perform better at the extremes, and discrepancies between formulae can themselves change risk classification, which is an important caveat when interpreting drug studies and individual tracings.^12^ Widely cited thresholds define a prolonged QTc as greater than 450 ms in men and greater than 470 ms in women, with values above 500 ms conferring the highest risk of TdP. Borderline and age- and sex-specific reference ranges are summarized in *Table 1*.^13^ A drug-induced increase of more than 60 ms from baseline is generally regarded as clinically meaningful even when the absolute QTc remains below 500 ms.^9,14^

**Table 1:** Heart-rate-corrected QT (QTc) interval reference ranges by age and sex

Age and sex	Normal QTc	Borderline QTc	Prolonged QTc
Children (<15 years)	<440 ms	440–460 ms	>460 ms
Adult men	<430 ms	430–450 ms	>450 ms
Adult women	<450 ms	450–470 ms	>470 ms

A QTc >500 ms or an increase >60 ms from baseline confers the highest risk of torsades de pointes. Adapted from Goldenberg et al. and Drew et al.^12,13^

QTc, heart-rate-corrected QT interval.

The concept of ‘repolarization reserve’ explains why some individuals tolerate QT-prolonging drugs while others develop TdP at modest exposures. Multiple, partially redundant outward currents normally safeguard repolarization; when this reserve is eroded by genetic variants, hypokalaemia, hypomagnesemia, bradycardia, or another QT-prolonging drug, the loss of a single current (typically IKr) can disproportionately prolong the action potential.^15,16^ Excessive prolongation permits reactivation of inward currents, generating early afterdepolarizations (EADs). In the presence of increased transmural dispersion of repolarization, these EADs provide both trigger and substrate for TdP.^3,15^ TdP is characteristically pause-dependent, often initiated after a short-long-short sequence following a premature ventricular beat, and may produce ventricular rates exceeding 200 beats per minute with abrupt haemodynamic collapse before either self-terminating or degenerating into VF.^17^

## Inherited (congenital) long QT syndrome

LQTS may be inherited or acquired, and the two forms exist on a mechanistic continuum rather than as wholly separate entities.^6,16^ This section addresses inherited (congenital) LQTS; acquired, drug-induced QT prolongation, the principal focus of this review, is addressed in Section 4. Inherited LQTS arises from pathogenic variants in genes encoding cardiac ion channels or their regulatory proteins, with more than 15 genotypes described. Three subtypes account for most genotype-positive cases: LQT1 (loss-of-function KCNQ1 variants that reduce IKs), LQT2 (KCNH2/hERG variants that reduce IKr), and LQT3 (gain-of-function SCN5A variants that enhance the late sodium current). Genotype influences the trigger for events. LQT1 events typically occur during exertion, especially swimming; LQT2 events are classically provoked by emotional stress or auditory stimuli, and LQT3 events tend to occur at rest or during sleep.^17,18^

Once considered rare, inherited LQTS is more common than long assumed, and a prospective ECG screening study of more than 44 000 infants estimated a prevalence of at least 1 in 2000 apparently healthy live births, making it one of the more frequent Mendelian disorders and a realistic, if often silent, presence in everyday practice.^19^ Clinically, inherited LQTS is divided into the autosomal dominant Romano-Ward syndrome and the rarer autosomal recessive Jervell and Lange-Nielsen syndrome, the latter associated with congenital sensorineural deafness and a more severe phenotype; in both subtypes the heart is typically structurally normal.^18^ The clinical spectrum ranges from lifelong asymptomatic QT prolongation to palpitations, syncope, seizures, and aborted cardiac arrest, with T-wave and U-wave abnormalities commonly seen on the ECG.^17^ Penetrance is incomplete, and expressivity is variable, where the resting QTc may be normal or only borderline in a substantial minority of genotype-positive individuals, yet syncope or cardiac arrest can still occur. Hence, a normal resting QTc does not exclude vulnerability, and a careful personal and family history is essential before exposing such patients to QT-prolonging antiemetics.^17,19^ Confirmed inherited LQTS mandates avoidance of QT-prolonging drugs as a cornerstone of management, alongside beta-blockade and, in selected cases, device therapy.^13,18^

## Acquired (drug-induced) QT prolongation: definition, mechanisms, and clinical implications

Acquired (drug-induced) LQTS is far more common in everyday practice and is, by definition, potentially preventable. It occurs when a medication, often layered on top of additional risk factors, causes the prolongation of repolarization sufficiently to provoke arrhythmia.^3^ A meaningful proportion of patients who develop drug-induced TdP harbour subclinical channelopathy variants or otherwise reduced repolarization reserve, which is why the same drug at the same dose is innocuous in most recipients yet catastrophic in a few.^6,16^ Class IA and III antiarrhythmics are the prototypical offenders, but a large and growing list of non-cardiac drugs, including antimicrobials (notably macrolides, fluoroquinolones, and azole antifungals), antipsychotics, methadone, antidepressants, and antiemetics among them, all can contribute substantially to the burden of acquired QT prolongation.^3^ Although the mechanisms described below apply broadly to any QT-prolonging drug, this review focuses specifically on antiemetics, a drug class that is ubiquitous across specialties and frequently co-administered with other torsadogenic agents, particularly in oncology.

The unifying mechanism of drug-induced QT prolongation is block of IKr. The hERG channel pore is unusually promiscuous, with a large inner cavity lined with aromatic residues that allows structurally diverse molecules to enter and bind, reducing repolarizing potassium efflux, prolonging the action potential, and lengthening the QT interval.^3,11,20^ This structural vulnerability explains why so many chemically unrelated drugs share a QT signal and why high-throughput hERG screening is central to preclinical cardiac safety testing.^6^

Importantly, IKr block is not the whole story. Some agents prolong repolarization by enhancing the late (persistent) inward sodium current during the plateau, which is mechanistically analogous to LQT3, and while others act indirectly through intracellular signalling, several anticancer agents, for example, prolong the QT interval through pathways distinct from direct channel block and are a growing concern as they are co-administered with antiemetics.^8,20^ These additive effects are especially relevant in oncology, where antiemetics accompany cytotoxic and targeted agents that themselves perturb repolarization.^7,8^ Whatever the molecular entry point, the final common pathway is the same, with excessive prolongation of repolarization, generation of EADs, and TdP.

## Neurophysiology of nausea and vomiting

Rational antiemetic selection is guided by the neurochemistry of emesis. The vomiting reflex is coordinated by a distributed network in the medulla, the ‘vomiting center’ comprising the nucleus tractus solitarius and the adjacent reticular formation, which integrates afferent input from four principal sources: the chemoreceptor trigger zone (CTZ) in the area postrema, the vestibular apparatus, the gastrointestinal tract via vagal afferents, and higher cortical and limbic centres.^1,21^ The CTZ lies on the floor of the fourth ventricle outside the blood-brain barrier, allowing it to sample emetogenic toxins, metabolites, and drugs circulating in the blood and cerebrospinal fluid; efferent signals then travel via cranial nerves V, VII, IX, andX and spinal pathways to the gastrointestinal tract, diaphragm, and abdominal musculature.^21^

Clinically, CINV is defined by its temporal relationship to chemotherapy and subdivided into acute (within 24 h of administration), delayed (24 h to 5 days), anticipatory (a conditioned response preceding treatment), breakthrough (occurring despite guideline-concordant prophylaxis), and refractory (unresponsive to both prophylaxis and rescue therapy) phenotypes; risk is stratified chiefly by the emetogenic potential of the chemotherapy regimen (high, moderate, low, or minimal) together with patient factors such as younger age, female sex, low habitual alcohol use, and a history of motion sickness or hyperemesis gravidarum.^4,5^ PONV is similarly multifactorial: validated risk scores incorporate female sex, non-smoking status, a prior history of PONV or motion sickness, and anticipated postoperative opioid use to guide the intensity of prophylaxis, with combination therapy recommended once two or more risk factors are present.^4^

Each afferent pathway is enriched in specific receptors, which constitute the pharmacological targets of antiemetic therapy: serotonin (5-HT3), dopamine (D2), and neurokinin-1 (NK1) receptors predominate in the CTZ and vomiting centre. Histamine (H1) and muscarinic (M1) receptors dominate the vestibular pathway, and 5-HT3, D2, and NK1 receptors mediate peripheral gastrointestinal signalling, with enterochromaffin-cell serotonin release a key trigger in CINV.^1,2^ Because distinct triggers recruit distinct receptors, combination antiemetic regimens targeting complementary pathways are standard in highly emetogenic settings, while simultaneously raising the probability of additive QT effects, a tension at the heart of safe prescribing.^4,5^ The principal afferent pathways of emesis and their corresponding antiemetic receptor targets are summarized in *Figure 2*.

**Figure 2:**
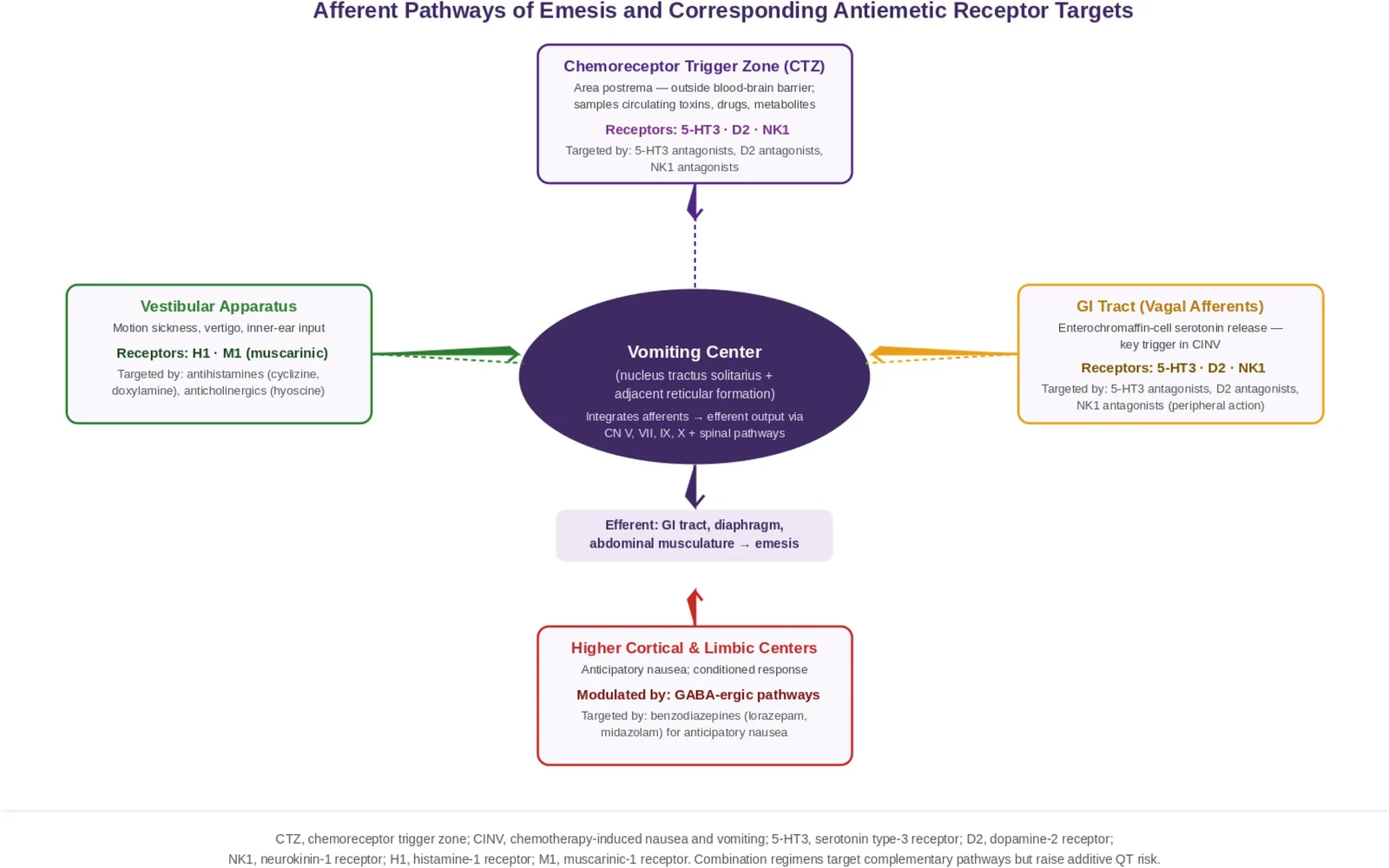
Afferent pathways of emesis and corresponding antiemetic receptor targets. The vomiting centre integrates input from the chemoreceptor trigger zone, the vestibular apparatus, gastrointestinal vagal afferents, and higher cortical/limbic centres, each enriched in distinct receptors that constitute the pharmacological targets of antiemetic therapy.

## Antiemetic drug classes and their QT liability

### Serotonin (5-HT3) receptor antagonists

The 5-HT3 antagonists: ondansetron, granisetron, dolasetron, tropisetron, and the second-generation agent palonosetron, are first-line therapy for CINV and PONV and act at both central (CTZ) and peripheral (vagal) 5-HT3 receptors.^4,5^ QT prolongation is a recognized class effect mediated chiefly by IKr block, but its magnitude differs substantially among agents. In a foundational electrophysiologic study of the class, ondansetron produced the most pronounced, concentration-dependent IKr blockade, whereas granisetron and dolasetron also blocked cardiac sodium channels, affecting depolarization and repolarization.^22^

Ondansetron carries the strongest regulatory and real-world signal. In 2012, following a dedicated, thorough QT study, the FDA warned that a single 32 mg intravenous dose produced clinically significant, dose-dependent QT prolongation. This dose was withdrawn from the label, and no single intravenous dose exceeding 16 mg is now recommended.^23^ Even standard intravenous doses produce measurable QTc increases, and low doses have precipitated TdP and cardiac arrest in susceptible individuals with electrolyte disturbance.^24^ A 2025 review focused on ondansetron in non-congenital LQTS concluded that serious events are concentrated among patients with inherited LQTS, electrolyte depletion, bradycardia, or concurrent QT-prolonging therapy rather than in the general population.^25^ In a 2026 disproportionality analysis of the FDA Adverse Event Reporting System (FAERS), ondansetron generated a markedly stronger QT-related signal [reporting odds ratio (ROR) 27.2] than olanzapine (ROR 8.7), consistent with their respective electrophysiologic profiles.^26^

Palonosetron, a second-generation 5-HT3 antagonist with higher receptor-binding affinity, allosteric interactions, and a substantially longer half-life, has minimal effects on the QT interval and is a preferred option when QT safety is paramount.^10,25^ Granisetron has a smaller QTc effect than ondansetron in a head-to-head comparison, and transdermal granisetron is of particular interest because it delivers a pharmacologically effective dose over several days without reaching the supratherapeutic peaks associated with bolus dosing, which is an attractive profile for at-risk patients.^27,28^ The 5-HT4 receptor agonists mosapride and prucalopride, used as prokinetics, enhance gastrointestinal motility without meaningful QT prolongation and may complement 5-HT3 blockade.^10^

### Neurokinin-1 (NK1) receptor antagonists

Aprepitant and its intravenous prodrug fosaprepitant, together with netupitant (usually co-formulated with palonosetron) and rolapitant, block substance P at central and peripheral NK1 receptors and are guideline-recommended additions to 5-HT3 antagonist plus dexamethasone regimens for highly and moderately emetogenic chemotherapy.^5^ A dedicated thorough-QT study in healthy subjects demonstrated that neither aprepitant nor fosaprepitant prolongs the QTc interval, confirming the class as electrophysiologically benign and particularly valuable for patients with QT concerns.^29^ A practical caveat is pharmacokinetic: aperitant is a moderate CYP3A4 inhibitor and can increase exposure to co-administered substrates, so interaction screening remains prudent, even though the NK1 class itself does not directly prolong the QT interval.^10^

### Dopamine (D2) antagonists: phenothiazines, butyrophenones, and benzamides

Dopamine antagonists act primarily at D2 receptors in the CTZ and comprise the phenothiazines (e.g. prochlorperazine, promethazine), butyrophenones (droperidol, haloperidol), and substituted benzamides (metoclopramide, domperidone). As a group they carry a comparatively high QT liability driven by combined D2 and IKr blockade.^10^ Droperidol received an FDA boxed warning in 2001 after reports of QT prolongation, TdP, and death; in a controlled comparison for PONV, low-dose droperidol produced a greater QTc increase than ondansetron, although both agents prolonged the QTc, and low antiemetic doses of droperidol continue to be used effectively with attention to cofactors and ECG awareness.^28^ Haloperidol similarly prolongs the QTc and has precipitated TdP, particularly with intravenous administration, higher doses, and concurrent electrolyte disturbance.^10^

Among the benzamides, domperidone has been linked in observational and pharmacovigilance studies to ventricular arrhythmia and sudden cardiac death and in a national pharmacovigilance case/non-case analysis, the reporting odds ratio for QT-related cardiac events was 2.0 for domperidone, 1.8 for ondansetron, and 3.4 for granisetron, findings that prompted dose and duration restrictions in several jurisdictions.^30^ Metoclopramide carries a lower but non-negligible risk and should be used at the lowest effective dose and for the shortest possible duration.^10^ Collectively, these agents are best reserved for patients without significant QT risk.

### Antihistamines and anticholinergics

First-generation H1 antihistamines (e.g. cyclizine, doxylamine, diphenhydramine, hydroxyzine, promethazine) are mainstays for motion sickness, vestibular nausea, and pregnancy-associated nausea (where doxylamine, often combined with pyridoxine, is first-line). Their QT profiles diverge meaningfully within the class, where promethazine significantly prolongs the QTc, even more so than the benzodiazepine midazolam in comparative data, while diphenhydramine and hydroxyzine prolong QT mainly at elevated or frankly toxic doses, and cyclizine and doxylamine carry little QT liability at therapeutic doses.^10^ The anticholinergic agents hyoscine (scopolamine, available as a transdermal patch) and trimethobenzamide are not associated with meaningful QT prolongation. All of these agents share sedative and anticholinergic adverse effects, including drowsiness, dry mouth, blurred vision, urinary retention, and, importantly in older adults, confusion and delirium. All of which should be weighed alongside their cardiac safety advantages.

### Atypical antipsychotics: olanzapine

Olanzapine has become an established component of antiemetic regimens for highly emetogenic chemotherapy, breakthrough CINV, and refractory nausea in palliative care, where its multireceptor antagonism (D2, 5-HT3, H1, and muscarinic) confers broad efficacy.^5,10^ As a second-generation antipsychotic, it produces less QT prolongation than first-generation agents and has among the smallest QTc effects within its class, but the effect is real and dose-dependent.^26^ In a prospective study of a domperidone, ondansetron, and olanzapine-containing regimen in patients with malignancy, olanzapine prolonged the QTc interval, and the combination produced further elevation, with some patients exceeding 480 ms, illustrating the additive hazard of multidrug regimens. Pragmatic thresholds derived from such data suggest dose reduction or substitution when the QTc exceeds 440 ms in men or 470 ms in women, and discontinuation above 500 ms.^31^ Real-world pharmacovigilance confirms a genuine but lower QT signal for olanzapine than for ondansetron, with risk amplified by the metabolic stress, electrolyte shifts, and polypharmacy typical of oncology populations.^26^

### Other agents: benzodiazepines, corticosteroids, and cannabinoids

Benzodiazepines (e.g. lorazepam, midazolam) and corticosteroids (dexamethasone) are valuable adjuncts that do not prolong the QT interval. Benzodiazepines are especially useful for anticipatory nausea, and dexamethasone is the backbone of CINV and PONV prophylaxis.^4,5^ Cannabinoid receptor agonists (e.g. nabilone, dronabinol) have a limited, second-line role and a modest cardiovascular profile, and are best positioned within a regimen that minimizes cumulative QT liability, reserving the higher-risk dopamine antagonists for refractory cases.^10^

## Evidence from trials, pharmacovigilance, and case series

Direct comparative trials reinforce the intra-class heterogeneity described above. In a prospective, single-blind, randomized trial in patients undergoing breast cancer surgery, intravenous ondansetron (8 mg) and granisetron (1 mg) both increased QTc from baseline within minutes, with the effect persisting for up to 1 h. However, the prolongation was greater with ondansetron; a significantly higher proportion of ondansetron-treated patients crossed the 440 ms threshold (approximately 59% vs. 33%), and the between-group difference was significant at 5 and 15 min, while granisetron-associated prolongation tended to attenuate at later time points.^32^ In a separate randomized PONV study, both droperidol and ondansetron measurably prolonged the QTc, with a greater effect from droperidol, reinforcing that QT liability is shared but graded across antiemetic classes and supporting granisetron and, especially, palonosetron when QT safety is a priority.^25,28^

Combination-regimen studies highlight the importance of additive effects. In a prospective, assessor-blinded study of a domperidone, ondansetron, and olanzapine-containing regimen, sequential exposure produced progressive QTc prolongation, with some patients exceeding 480 ms, demonstrating that the risk classification and number of co-administered torsadogenic agents, rather than any single drug, drive cumulative hazard.^31^ Large-scale pharmacovigilance corroborates these observations, including a disproportionality analysis of FAERS, which identified ondansetron among the drugs most frequently implicated in fatal drug–drug-interaction-associated TdP, often combined with antidepressants, antipsychotics, methadone, fluoroquinolones, or azole antifungals.^33^ National pharmacovigilance data similarly associate domperidone, ondansetron, and granisetron with QT-related cardiac events.^30^

Case reports anchor the population-level data in individual physiology. Multiple reports describe TdP and cardiac arrest after standard, single oral, or 4 mg intravenous doses of ondansetron, almost invariably in patients with hypokalaemia, hypomagnesemia, bradycardia, or concomitant QT-prolonging drugs, with QT intervals normalizing once the offending agent and cofactors are removed.^24^ The recurring lesson is consistent: antiemetic-associated TdP rarely results from a single drug acting on a normal heart; more often, it reflects the convergence of an agent with one or more modifiable risk factors.

## Risk factors and drug–drug interactions

Susceptibility to antiemetic-associated TdP reflects the cumulative burden of patient- and drug-related factors, and these have been formalized into a validated bedside risk score. In hospitalized patients, independent predictors of QTc prolongation (defined as QTc >500 ms or an increase of >60 ms from baseline) include female sex, an admitting QTc above 450 ms, myocardial infarction, septic shock, left ventricular dysfunction, serum potassium below 3.5 mEq/L, loop diuretic use, age over 68 years, and administration of one or, especially, two or more QT-prolonging drugs. The resulting score discriminates well between high-risk patients (C-statistic 0.82) and operationalizes the risk assessment that should precede the use of higher-risk antiemetics.^14^ Additional non-modifiable contributors include inherited LQTS or a family history of sudden cardiac death and structural heart disease.^9^

Modifiable and situational factors include hypokalaemia, hypomagnesemia, and hypocalcaemia (frequently produced by the very vomiting being treated), bradycardia, hepatic or renal impairment that raises drug exposure, rapid intravenous administration, and high or cumulative dosing.^3,14^ Polypharmacy deserves particular emphasis in oncology and palliative care, where antiemetics accompany cytotoxic agents, kinase inhibitors, antifungals, fluoroquinolones, methadone, and antidepressants.^8,10^ FAERS analyses confirm that the risk classification of co-administered agents and not merely their number, best predicts TdP, and that antiemetics frequently appear in the highest-risk interaction pairs.^33^ Pharmacokinetic interactions compound the problem, where drugs that inhibit hepatic metabolism (for example, CYP2D6 or CYP3A4 inhibitors) can push a QT-prolonging antiemetic into a more torsadogenic concentration range. Curated resources, such as the CredibleMeds/AZCERT QTdrugs list (https://www.crediblemeds.org), which stratifies medicines by the known, possible, and conditional risk of TdP, are practical screening tools at the point of prescribing.^9,34^

## A risk-stratified approach to antiemetic selection

In patients with congenital or acquired LQTS, or with multiple torsadogenic risk factors, antiemetic therapy should follow a deliberate, risk-based sequence rather than default to the most familiar agent. We propose a three-tier classification (*Tables 2* and *3*) IKr, rapid delayed rectifier potassium current; hERG, human ether-a-go-go-related gene; D2, dopamine-2 receptor; H1, histamine-1 receptor; NK1, neurokinin-1 receptor; IV, intravenous. Liability reflects a synthesis of mechanistic, trial, and pharmacovigilance data.^10,22,30,31^ and an accompanying stepwise algorithm (*Figure 3*). First-line agents are those with minimal or no QT liability and are preferred in high-risk settings: the NK1 antagonists (aprepitant, fosaprepitant), the low-risk first-generation antihistamines (cyclizine, doxylamine), anticholinergics (hyoscine, trimethobenzamide), benzodiazepines (lorazepam, midazolam), corticosteroids (dexamethasone), the 5-HT4 prokinetics (mosapride, prucalopride), and among 5-HT3 antagonists, palonosetron.^25,29^ Second-line agents carry intermediate risk and warrant caution and monitoring, including higher-dose or parenteral diphenhydramine and hydroxyzine, metoclopramide, and olanzapine.^10,31^ Third-line agents carry the greatest QT liability and should be reserved for situations where benefits clearly outweigh risks or alternatives have failed, such as promethazine, the butyrophenones (droperidol, haloperidol) and domperidone, and the 5-HT3 antagonists granisetron and dolasetron at higher exposures, with intravenous ondansetron used cautiously and within dose limits.^30^

**Figure 3:**
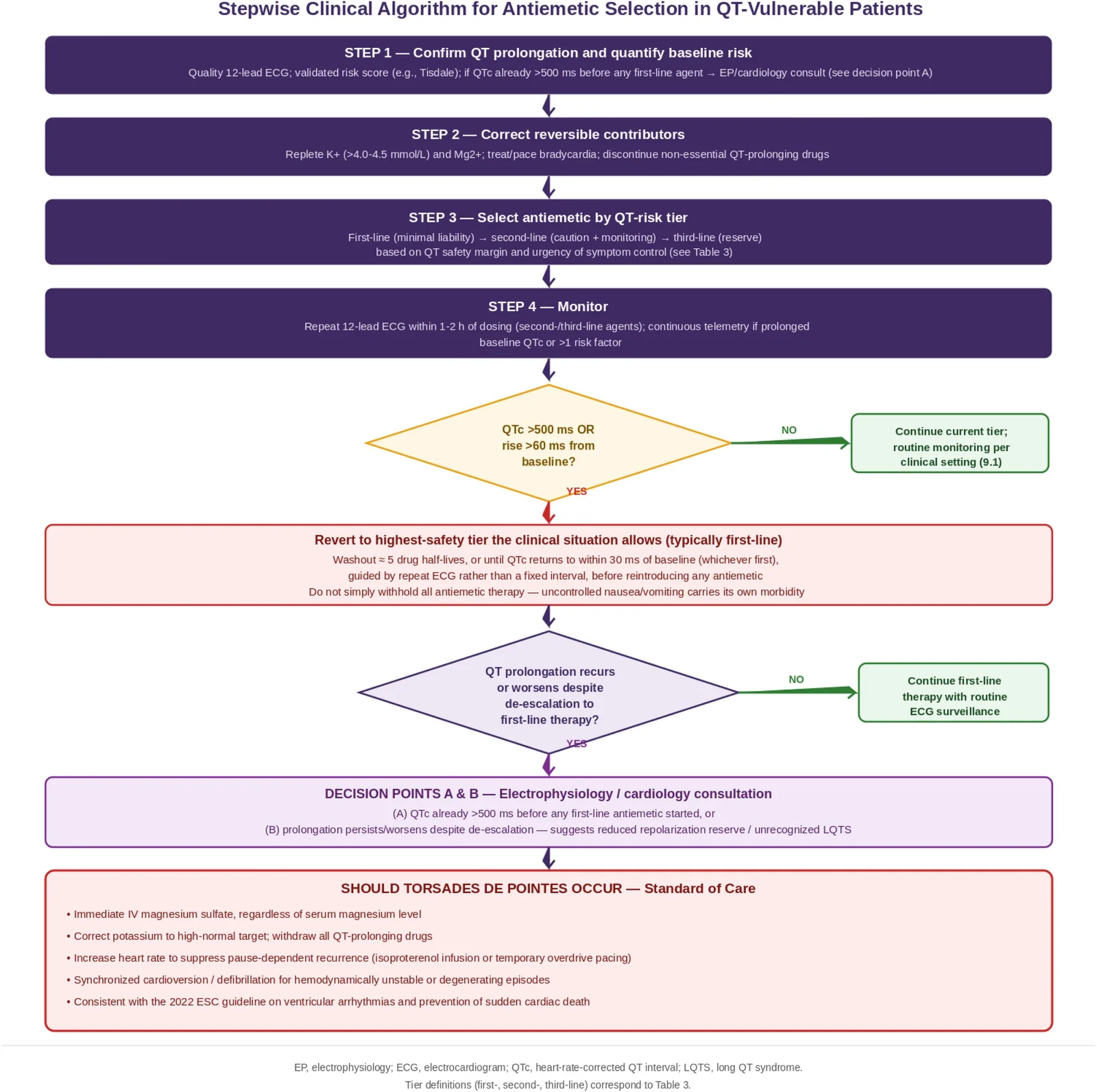
Stepwise clinical algorithm for antiemetic selection in patients with QT prolongation. After confirming QT prolongation and quantifying baseline risk, reversible causes (electrolyte depletion, bradycardia, non-essential QT-prolonging drugs) are corrected before progressing through first-, second-, and third-line options based on QT safety (see *Table 3*). If QTc exceeds 500 ms or rises more than 60 ms from baseline on a second- or third-line agent, the algorithm reverts to the highest-safety tier with a defined washout, with electrophysiology or cardiology consultation at two decision points and a defined standard of care should torsades de pointes occur.

**Table 2:** QT-interval liability of antiemetic drug classes

Class	Representative agents	Principal mechanism	QT liability
5-HT3 antagonists	Ondansetron, granisetron, dolasetron, palonosetron	IKr (hERG) block; Na+block (granisetron, dolasetron)	High (IV ondansetron) to minimal (palonosetron)
NK1 antagonists	Aprepitant, fosaprepitant, netupitant	Substance P/NK1 blockade	Minimal
Phenothiazines	Prochlorperazine, promethazine	D2 (and H1) blockade; IKr block	Moderate-high
Butyrophenones	Droperidol, haloperidol	D2 blockade; IKr block	High (dose-dependent)
Benzamides	Metoclopramide, domperidone	D2 blockade; IKr block	Moderate (metoclopramide); high (domperidone)
H1 antihistamines	Cyclizine, doxylamine, diphenhydramine, hydroxyzine, promethazine	H1 (and muscarinic) blockade; IKr block at high dose	Minimal (cyclizine, doxylamine) to high (promethazine)
Anticholinergics	Hyoscine, trimethobenzamide	Muscarinic (M1) blockade	Minimal
Atypical antipsychotic	Olanzapine	Multireceptor; modest IKr block	Low-moderate (dose-dependent)
5-HT4 agonists	Mosapride, prucalopride	Prokinetic 5-HT4 agonism	Minimal
Adjuncts	Dexamethasone, lorazepam, midazolam	Corticosteroid; GABA-ergic	Negligible

IKr, rapid delayed rectifier potassium current; hERG, human ether-a-go-go-related gene; D2, dopamine-2 receptor; H1, histamine-1 receptor; NK1, neurokinin-1 receptor; IV, intravenous. Liability reflects a synthesis of mechanistic, trial, and pharmacovigilance data.^10,22,30,31^

**Table 3:** Risk-stratified (first-, second-, and third-line) antiemetic selection by QT safety

First line (minimal QT liability)	Second line (intermediate; caution + monitoring)	Third line (high QT liability; reserve)
**NK1 antagonists:** Aprepitant, fosaprepitant **H1 antihistamines:** Cyclizine, doxylamine **Anticholinergics:** Hyoscine, trimethobenzamide **5-HT3 antagonist:** Palonosetron **5-HT4 agonists:** Mosapride, prucalopride **Adjuncts:** Dexamethasone, lorazepam, midazolam	**H1 antihistamines:** Diphenhydramine, hydroxyzine **D2 antagonist:** Metoclopramide **Atypical antipsychotic:** Olanzapine	**H1 antihistamine:** Promethazine **5-HT3 antagonists:** Granisetron, dolasetron; IV ondansetron (within dose limits) **Butyrophenones/benzamide:** Droperidol, haloperidol, domperidone

Tiers reflect QT/torsadogenic liability, not antiemetic efficacy. Selection always follows correction of reversible causes (electrolytes, bradycardia, non-essential QT-prolonging drugs). See *Figure 3* for the stepwise algorithm.

Critically, drug selection is the second step, not the first. The algorithm begins by confirming QT prolongation on a quality 12-lead ECG and quantifying baseline risk, then correcting reversible contributors, such as replenishing potassium (target greater than 4.0–4.5 mmol/L) and magnesium, treating bradycardia, and discontinuing non-essential QT-prolonging medications, all before escalating through the tiers based on the patient’s QT safety margin and the clinical urgency of symptom control.^9,14^ When the QTc exceeds 500 ms or rises more than 60 ms from baseline on a second- or third-line agent, we recommend reverting to the highest-safety tier the clinical situation allows, typically a first-line agent, rather than simply withholding all antiemetic therapy, since uncontrolled nausea and vomiting carry their own morbidity. A practical washout of approximately five drug half-lives, or until the QTc has returned to within 30 ms of the patient’s baseline, whichever is achieved first, is reasonable before reintroducing any antiemetic, guided by repeat ECG rather than a fixed interval alone. Continuous telemetry, or a repeat 12-lead ECG within 1–2 h of dosing, is advisable whenever a second- or third-line agent is used in a patient with a prolonged baseline QTc or more than one risk factor. Electrophysiology or cardiology consultation is warranted at two distinct points: when the QTc is already substantially prolonged (greater than 500 ms) before any first-line antiemetic has even been started, and when QT prolongation occurs or worsens despite stepwise de-escalation to first-line therapy, since the latter suggests a lower repolarization reserve than anticipated and may indicate previously unrecognized LQTS. Should TdP nonetheless occur, standard of care includes immediate intravenous magnesium sulphate regardless of serum magnesium level, correction of potassium to high-normal targets, withdrawal of all QT-prolonging drugs, and measures to increase heart rate (isoproterenol infusion or temporary overdrive pacing) to suppress pause-dependent recurrence; synchronized cardioversion or defibrillation is required for haemodynamically unstable or degenerating episodes.^13^ This is consistent with the 2022 ESC guideline on the management of ventricular arrhythmias and the prevention of sudden cardiac death.^35^ If all pharmacologic options are exhausted or contraindicated, electrophysiology or specialist consultation is recommended.^9^

This framework builds on and extends a previously proposed decision-support flowchart for QT-prolonging anticancer and antiemetic drugs, which classified individual agents by QT risk but did not integrate systematic reversible-cause correction, explicit escalation and de-escalation thresholds, or specialist-consultation triggers into a single, stepwise bedside algorithm.^36^ The framework proposed here (*Figure 3*) operationalizes these elements explicitly and extends applicability beyond oncology to any patient receiving antiemetics who has LQTS or other torsadogenic risk factors.

### Practical notes by clinical setting

The framework adapts across settings. In oncology (CINV), guideline-concordant prophylaxis combines an NK1 antagonist, a 5-HT3 antagonist, dexamethasone, and increasingly olanzapine for highly emetogenic regimens; in QT-vulnerable patients, palonosetron is the preferred 5-HT3 component, and electrolyte surveillance is essential given concomitant cytotoxic exposure.^5,8^ Guideline-concordant prophylaxis in this setting also aligns with the 2026 NCCN Antiemesis guideline.^37^ The 2023 MASCC/ESMO update reaffirms this structure and, together with a 2025 gap analysis, highlights lower-than-standard olanzapine doses (<10 mg/day) as an emerging option for limiting cumulative sedative and QT-relevant burden without sacrificing antiemetic efficacy.^38,39^ Consistent with the 2022 ESC cardio-oncology guideline’s recommendation for baseline and on-treatment QTc surveillance in patients receiving QT-prolonging anticancer therapy, electrolyte and ECG monitoring should accompany antiemetic selection throughout the treatment course, not only at initiation.^40^ In the perioperative period (PONV), multimodal prophylaxis with agents acting at different receptors is standard, and substituting lower-risk agents (dexamethasone, an NK1 antagonist, or palonosetron) for higher-risk choices is straightforward when the QTc is already prolonged.^4^ In the emergency department, where ondansetron is widely used and baseline ECGs and electrolytes are often unavailable, vigilance for occult risk factors is warranted, as TdP has occurred even with single low doses.^24^ Formal risk scoring can guide which patients warrant a baseline ECG and telemetry before higher-risk therapy.^14^

## Monitoring and regulatory context

The regulatory architecture governing QT safety is grounded in the ICH E14 guideline, which standardized the thorough QT/QTc study for non-antiarrhythmic drugs, and its nonclinical companion S7B; together, they made systematic QT assessment a routine component of drug development.^3,8^ The ondansetron experience exemplifies how post-marketing surveillance and dedicated QT studies translate into label changes, as with the 2012 withdrawal of the 32 mg intravenous dose.^22,23^ In practice, prudent monitoring for at-risk patients includes a baseline 12-lead ECG and serum electrolytes (potassium, magnesium, and calcium) before initiating higher-risk antiemetics. Repeat ECGs after dosing or dose escalation in monitored settings, continuous telemetry when multiple risk factors coexist, correction of electrolytes to high-normal targets, and ready availability of intravenous magnesium for the treatment of TdP.^13^ Validated risk scores and curated drug lists help target monitoring to the patients most likely to benefit and reduce inadvertent high-risk combinations.^9,14^

## Limitations and future directions

Several caveats temper the current evidence. Much of the literature linking antiemetics to TdP derives from case reports, small physiologic studies, and disproportionality analyses of spontaneous-reporting databases, such as FAERS, which establish association and signal strength but are subject to reporting bias and cannot quantify absolute incidence or prove causation.^26,33^ Reported QTc values are sensitive to the rate-correction formula and to measurement conditions, complicating cross-study comparison.^12^Randomized data directly comparing the arrhythmic safety of antiemetics in high-risk populations remains sparse. Prospective, adequately powered studies, particularly in patients with genotyped inherited LQTS and those on complex oncologic regimens, are needed to quantify absolute risk and to validate risk-stratified frameworks.^19^ Pharmacogenomic profiling of repolarization reserve, automated QT surveillance integrated into the electronic health record, and refinement of validated risk scores represent promising avenues for personalizing antiemetic therapy.^14,16^ Wider, guideline-directed use of genetic testing and cascade screening in relatives of an LQTS proband could also help identify at-risk individuals before an antiemetic exposure precipitates the first arrhythmic event, although a multinational survey found access to cardiac genetic testing highly heterogeneous and frequently limited by laboratory availability and reimbursement.^41,42^ Preclinical tools, such as the hERG IC50:Cmax ratio, which correlates with clinical TdP incidence across drug classes, may further help translate channel-level data into individualized risk estimates as these frameworks mature.^43^

## Conclusion

Antiemetics are indispensable, but several classes share an ability to delay ventricular repolarization and, in vulnerable patients, to precipitate TdP and sudden death. The risk is heterogeneous within as well as between classes, and it is overwhelmingly concentrated in patients with electrolyte disturbance, bradycardia, polypharmacy, or inherited LQTS. Because these cofactors are largely modifiable, antiemetic-associated arrhythmia is, for the most part, preventable. A disciplined approach that includes a structured risk assessment using validated tools, electrolyte correction, judicious agent selection guided by the classification and algorithm presented here, and appropriate ECG monitoring allows clinicians to control nausea and vomiting effectively while safeguarding the heart’s electrical stability. As antiemetic combinations grow more complex, particularly in oncology, this risk-based mindset will only become more important.^4,14^

## Supplementary Material

pvag077_Supplementary_Data

## Data Availability

No new data were generated or analysed in support of this research. The data were collected from published articles listed in the references.
