# Supplementary material for: Antiemetic-associated QT prolongation: a clinical review and risk-stratified prescribing framework for long QT syndrome
Source: Eur Heart J Cardiovasc Pharmacother. 2026 Sep 16;12(7):556–67. doi: 10.1093/ehjcvp/pvag077 (PMC13730583; doi:10.1093/ehjcvp/pvag077)
Supplement: Supplementary file 1 [file pvag077_supplementary_data.docx]

**Supplementary Material**

Search Strategy — "Antiemetic-Associated QT Prolongation: A Clinical Review and Risk-Stratified Prescribing Framework for Long QT Syndrome" (Manuscript EHJ-CVP-D-26-00354)

**1. Databases and Date Range**

PubMed/MEDLINE and EMBASE were searched from January 1, 2000 to February 28, 2026. Reference lists of all retrieved articles, relevant clinical practice guidelines, and consensus statements were hand-searched for additional eligible studies. No language restriction was applied at the search stage; non-English records without an available English abstract were excluded at screening.

**2. Search Terms**

The following MeSH terms and free-text keywords were combined using Boolean operators (AND/OR), searched as both subject headings and title/abstract fields where applicable:

- "Antiemetics"[Mesh] OR antiemetic* OR "anti-emetic*"
- "Serotonin 5-HT3 Receptor Antagonists"[Mesh] OR "5-HT3 receptor antagonist*" OR ondansetron OR granisetron OR dolasetron OR tropisetron OR palonosetron
- "Dopamine Antagonists"[Mesh] OR "dopamine antagonist*" OR metoclopramide OR domperidone OR droperidol OR haloperidol OR prochlorperazine OR promethazine
- "Neurokinin-1 Receptor Antagonists"[Mesh] OR "NK1 antagonist*" OR aprepitant OR fosaprepitant OR netupitant OR rolapitant
- olanzapine OR "atypical antipsychotic*"
- "Long QT Syndrome"[Mesh] OR "long QT syndrome" OR LQTS OR "QT prolongation" OR "QTc prolongation"
- "Torsades de Pointes"[Mesh] OR "torsades de pointes" OR TdP OR "ventricular fibrillation" OR "sudden cardiac death"
- "drug-induced arrhythmia*" OR "drug-induced long QT" OR "acquired long QT"
- "chemotherapy-induced nausea and vomiting" OR CINV OR "postoperative nausea and vomiting" OR PONV
- "pharmacovigilance" OR "FDA Adverse Event Reporting System" OR FAERS OR "disproportionality analysis" OR "reporting odds ratio"

**3. Supplementary and Grey-Literature Sources**

The CredibleMeds/AZCERT QTdrugs list was consulted to cross-check the torsadogenic risk classification of individual agents. Current clinical practice guidelines were retrieved directly from the websites of the issuing societies, including the American Society of Clinical Oncology (ASCO), the Multinational Association of Supportive Care in Cancer/European Society for Medical Oncology (MASCC/ESMO), the National Comprehensive Cancer Network (NCCN), the American Heart Association/American College of Cardiology, the European Society of Cardiology (ESC), and the European Heart Rhythm Association (EHRA)/Heart Rhythm Society (HRS).

**4. Study Selection: Inclusion and Exclusion Criteria**

Included: mechanistic/electrophysiologic studies, randomized and non-randomized comparative trials, pharmacovigilance disproportionality analyses, systematic reviews, and consensus or guideline statements addressing QT-interval effects of antiemetic agents or the diagnosis and management of inherited or acquired long QT syndrome, published in English between 2000 and 2026.

Excluded: conference abstracts without a corresponding full-text publication, case reports describing agents or clinical contexts outside the scope of antiemetic prescribing, and articles for which the full text could not be retrieved.

Case reports and small case series were included selectively, where they illustrated a mechanism or a clinically important signal not otherwise captured by larger studies (e.g., torsades de pointes after low-dose intravenous ondansetron).

**5. Evaluation of Pharmacovigilance (FAERS) Evidence**

Findings from FDA Adverse Event Reporting System (FAERS) disproportionality analyses were considered supportive when reported in a peer-reviewed publication with an adequate case count and a disproportionality measure (e.g., reporting odds ratio) accompanied by a confidence interval or equivalent measure of precision. Consistent with the inherent limitations of spontaneous-reporting pharmacovigilance data, these analyses were interpreted as evidence of association and signal strength rather than as evidence of causation or absolute incidence, and this limitation is discussed explicitly in Section 11 of the main text.

**6. Study Selection Process**

One author (H.R.K.) performed the primary search and initial title/abstract screening; disagreements or uncertain eligibility decisions were resolved by discussion among the author team. Given the narrative-review design, a formal PRISMA flow diagram was not generated; the search strategy described here is provided in full to support transparency and reproducibility.
